# Wavelength selective photoactivated autocatalytic oxidation of 5,12-dihydrobenzo[*b*]phenazine and its application in metal-free synthesis†

**DOI:** 10.1039/d0ra01495h

**Published:** 2020-03-09

**Authors:** Lei Bian, Jie Ma, Xiaotong Feng, Yuanhang Wang, Lizhi Zhao, Lei Zhao, Xiayan Wang, Guangsheng Guo, Qiaosheng Pu

**Affiliations:** State Key Laboratory of Applied Organic Chemistry, Key Laboratory of Nonferrous Metals Chemistry and Resources Utilization of Gansu Province, College of Chemistry and Chemical Engineering, Lanzhou University Lanzhou 730000 China puqs@lzu.edu.cn; College of Chemical Engineering and Technology, Tianshui Normal University Tianshui 741001 China; Department of Chemistry and Chemical Engineering, Beijing University of Technology Beijing 100124 China

## Abstract

Photochemical stability of 5,12-dihydrobenzo[*b*]phenazine (DHBP) was investigated with LEDs with central emission wavelengths in a range of 365 to 595 nm. Photochemical conversion of DHBP to benzo[*b*]phenazine (BP) was observed with wavelengths upto 516 nm. Light of 490 and 516 nm is not absorbed by DHBP, but photoactivated autocatalytic oxidation of DHBP to BP with these wavelengths was confirmed. The reaction rate is in a range of 111–208 μg min^−1^ with these LEDs. The mechanism of the reaction was examined and the experimental results exclude the intermolecular interaction such as the Förster resonance energy transfer, the intermolecular charge transfer, the photoinduced electron transfer and the formation of an exciplex. The formation of the reactive oxygen species was verified with electron paramagnetic resonance, which indicates its potential in the synthesis. When sunlight was used as the light source, the oxidation rate of 1 mg mL^−1^ DHBP was 393 μg min^−1^. Same autocatalytic oxidation was also observed on similar compounds and it can be used for producing metal-free organic substances for semiconductors.

## Abstract 2

We describe the photoactivated autocatalytic oxidation of 5,12-dihydrobenzo[*b*]phenazine to benzo[*b*]phenazine with oxygen from the air as the oxidant and develop a green synthesis way of metal impurity-free BP or other similar products.

## Graphical abstract

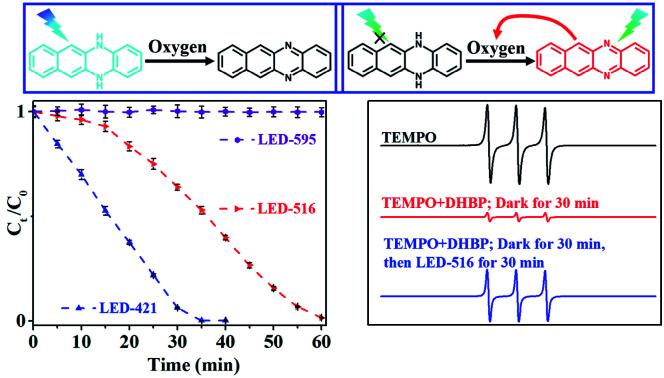

## 1. Introduction

**Scheme 1:**
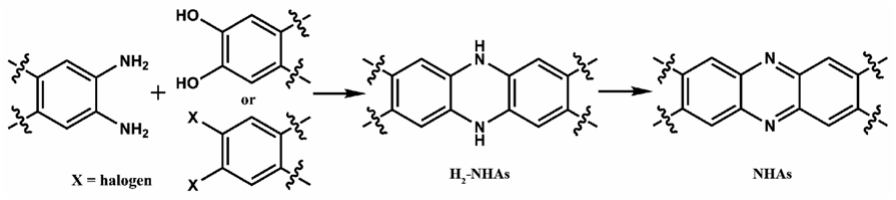
Synthesis of NHAs from aromatic *o*-diamines and aromatic *o*-dihydroxyarenes or activated *o*-dihalides.

*N*-Heteroacenes (NHAs) are a group of well-known molecules that are widely used in organic semiconductor devices.^1^ Their utilization in chemical/bio sensing,^2^ OLEDs (as the thermally activated delayed fluorescence organic light emitters)^3^ and the preparation of diradicals^4,5^ has also been reported. These molecules can be prepared through the condensation of aromatic *o*-diamines with aromatic *o*-dihydroxyarenes or activated *o*-dihalides,^6^ but the direct products of this condensation are dihydro *N*-heteroacene (H_2_-NHA) derivatives, which normally exhibit good chemical stability and are often used to produce other H_2_-NHA derivatives. To get NHAs, oxidation of the H_2_-NHA is frequently needed (Scheme 1). Oxidants, such as MnO_2_ (ref. 7–10) and potassium dichromate,^11^ are suitable for the oxidation of H_2_-NHAs to NHAs in most of cases. However, residual metal ions in the product can be a big concern in the semiconductor industry where the requirement of purity is normally pretty high. On the other hand, heavy metal compounds are also harmful to the environment and may be a threat to human health. It is reported that excess manganese can damage the nervous system and cause a series of symptoms, such as sleep disturbances, muscular pain and hypertonia, hallucinations, and mental irritability.^12^ The accumulation of Cr(vi) in the human body can lead to many diseases, such as skin allergy, kidney disease and cancer.^13^ Therefore, it is of great significance to develop metal-free green synthesis methods for the oxidation of the H_2_-NHAs.

NHAs exhibit some unique photochemical properties^14^ and their photochemistry deserves investigation. Photochemistry is common in nature, photosynthesis of oxygen by plants, isomerization of some organic molecules for the vision are all typical photochemical processes which adopt the energy of light. The importance of photochemistry in organic synthesis,^15,16^ CO_2_ reduction,^17,18^ and hydrogen production^19^ has again attracted wide attention recently. Photoactivated oxidation is an important reaction of photochemistry, and related researches will help us to find the substitutes of oxidants containing heavy metals. With a proper photocatalyst, metal-free active oxides with special oxidation ability can be produced in the system.^20^

Photobleaching is a well known photochemical phenomenon that is normally detrimental and is one of the shortcomings of many organic dyes. Some of photobleaching reactions have been proved to be related to the photoactivated oxidation.^21^ Photobleaching frequently produces complicated intermediates or by-products, barely be used for the synthetic purpose. Therefore, fully understand of the photochemical process of a photochemical substance is critical for its proper application. Photoactivated autocatalysis route may accelerate a photochemical reaction, where the product transfers the light energy to the reactants.^22^ If this type of energy transfer is smooth enough, it will efficiently prevent the damage of the product and ensure the complete conversion of the starting material.

As typical NHAs, phenazines, have been reported to be as photocatalysts in atom transfer radicals polymerization.^14,23^ However, comprehensive investigation of their own photochemical reaction has been scarcely reported. In this work, possible reactions of H_2_-NHAs were systematically investigated by using LEDs of different emission wavelengths. Photoactivated oxidation of 5,12-dihydrobenzo[*b*]phenazine (DHBP) to benzo[*b*]phenazine (BP) was confirmed for the wavelengths that are within the absorption band of DHBP. More importantly, oxidation of DHBP was also confirmed with irradiation wavelengths beyond the absorption edge of DHBP, which showed typical autocatalytic characteristics. This implied the possibility of using photons with lower energy for the reaction. The process can be developed as a green synthesis way of metal impurity-free BP or other similar products.

## 2. Experimental section

### 2.1 Materials and methods

Chemicals were purchased from commercial sources and used as received without further purification. 5,12-Dihydrobenzo[*b*]phenazine (DHBP) and 5,12-dihydro-2,3-dimethylbenzo[*b*]phenazine (DMDHBP) were prepared according to the literature procedures.^11^ The mixed solvent is a mixture of DMSO and ethanol in a volume ratio of 4 to 1.

LEDs and constant current source were purchased from Taobao (www.taobao.com). Two crocodile clips were soldered to the constant current source output to facilitate replacement of different LEDs. Working parameters of LEDs were showed in Fig. S8, Tables S1 and S2.†

The emission spectra of the LEDs were measured with a PMS-80 UV-vis-near IR Spectrophotocolorimeter (Everfine, Hangzhou, China). Fluorescence spectra were recorded on a F97Pro fluorospectrophotometer (Shanghai Lengguang Technology Co., Ltd., Shanghai, China), and UV-vis absorption spectra were measured using a UV 2800SPC spectrophotometer (Shanghai Sunny Hengping Scientific Instrument Co., Ltd., Shanghai, China). Nuclear magnetic resonance (NMR) spectra were obtained with a JNM-ECS spectrometer (JEOL, Tokyo, Japan, 400 MHz). Mass spectra were recorded on a microTOF mass spectrometer (Bruker Daltonics, Bremen, Germany) equipped with an electrospray ionization source. Electron paramagnetic resonance (EPR) spectra were recorded in quartz tubes on a Bruker A300-9.5/12 device. Experimental conditions: temperature 300 K, microwave frequency 9.445 GHz, microwave power 7.7 mW.

### 2.2 Photolysis and photoactivated autocatalytic oxidation of DHBP

Different concentrations of DHBP solution were prepared with the mixed solvent, and the fluorescence intensities of DHBP at 455 nm (*λ*_ex_ = 408 nm, Fig. S1†) were tested to obtain the relationship between fluorescence intensities and concentrations.

#### LED photocatalytic oxidation experiment

5 mL of DHBP at a concentration of 1 mg mL^−1^ was added to a weighing bottle (30 mm i.d.) that was placed on a water bath of 10 °C. The LED was arranged on the top of the weighing bottle to illuminate the reaction solution (35 mm above the surface of the solution). The solution was stirred with a 5 mm magnetic stir bar at a speed of 200 rpm. After a given period of time, 50 μL of aliquot was taken out from the bottle and diluted for the fluorescence measurement. The control experiment was similar to this procedure but without light irradiation. Experimental conditions including wavelength of the illumination, intensity of light, reaction temperature, initial concentration of DHBP, and atmosphere (N_2_, O_2_ or air) were investigated.

#### Sunlight catalytic oxidation

Sunlight (at noon) was used instead of LEDs as the light source. The other experimental operation was same as described above.

### 2.3 Synthesis

#### 5,12-Dihydrobenzo[*b*]phenazine (DHBP)^11^

Naphthalene-2,3-diol (320.0 mg, 2.0 mmol) and *o*-phenylenediamine (216.0 mg, 2.0 mmol) were mixed in an agate mortar and ground to a uniform powder. Then the mixture was transferred into a glass tube (10 mm × 75 mm) and heated under an atmosphere of nitrogen at 180 °C for 0.5 h. After cooled down to room temperature, the material was then washed on a filter with methanol, acetone and diethyl ether. The resulting material was collected and dried to give DHBP as a yellow solid (371.0 mg, 80%). ^1^H-NMR (*δ* in DMSO-d_6_) 8.05 (s, 2H), 7.14 (m, 2H), 6.89 (m, 2H), 6.34 (m, 2H), 6.23 (s, 2H), 6.16 (m, 2H). ^13^C-NMR (*δ* in DMSO-d_6_) 135.03, 132.17, 131.33, 125.29, 123.30, 120.69, 112.21, 104.81. MS (ESI) [M + H]^+^*m*/*z* = 233.1 (meas.), 233.1 (calc.).

#### 5,12-Dihydro-2,3-dimethylbenzo[*b*]phenazine (DMDHBP)^11^

Naphthalene-2,3-diol (80.0 mg, 0.5 mmol) and 4,5-dimethyl-1,2-phenylenediamine (68.1 mg, 0.5 mmol) were mixed in an agate mortar and ground to a uniform powder. Then the mixture was transferred into a glass tube (10 mm × 100 mm) and heated under an atmosphere of nitrogen at 180 °C for 0.5 h. After cooled down to room temperature, the material was then washed on a filter with methanol, acetone and diethyl ether. The resulting material was collected and dried to give DMDHBP (100.0 mg, 77%). ^1^H-NMR (*δ* in DMSO-d_6_) 7.89 (s, 2H), 7.13 (m, 2H), 6.88 (m, 2H), 6.21 (s, 2H), 5.99 (s, 2H), 1.92 (s, 6H). ^13^C-NMR (*δ* in DMSO-d_6_) 135.29, 131.25, 129.65, 127.30, 125.13, 123.10, 113.88, 104.53, 19.03. MS (ESI) [M + H]^+^*m*/*z* = 261.1 (meas.), 261.1 (calc.).

#### Benzo[*b*]phenazine (BP)

A solution of DHBP (5.0 mg, 21.6 μmol) in the mixed solvent (5 mL) was stirred in air under light irradiation using LED-391. When the fluorescence intensity at 455 nm fell to less than 1% of the initial value, an excess amount of water was added to the reaction solution to obtain precipitation. The resulting precipitate was separated, washed with water and dried. DHBP was almost completely converted into BP. ^1^H-NMR (*δ* in CDCl_3_) 8.92 (s, 2H), 8.25 (m, 2H), 8.13 (m, 2H), 7.82 (m, 2H), 7.54 (m, 2H). ^13^C-NMR (*δ* in CDCl_3_) 144.48, 139.95, 134.72, 130.97, 129.89, 128.67, 127.65, 127.08. MS (ESI) [M + H]^+^*m*/*z* = 231.1 (meas.), 231.1 (calc.).

#### 2,3-Dimethylbenzo[*b*]phenazine (DMBP)

A solution of DMDHBP (5.0 mg, 19.2 μmol) in the mixed solvent (5 mL) was stirred in air under light irradiation using LED-391. When the fluorescence intensity at 455 nm fell to less than 1% of the initial value, an excess amount of water was added to the reaction solution to obtain precipitation. The resulting precipitate was separated, washed with water and dried. DMDHBP was almost completely converted into DMBP. ^1^H-NMR (*δ* in CDCl_3_) 8.85 (s, 2H), 8.11 (m, 2H), 7.94 (s, 2H), 7.52 (m, 2H), 2.56 (s, 6H). ^13^C-NMR (*δ* in CDCl_3_) 135.14, 134.32, 129.90, 128.60, 128.29, 127.59, 127.20, 126.83, 20.98. MS (ESI) [M + H]^+^*m*/*z* = 259.1 (meas.), 259.1 (calc.).

## 3. Results and discussion

### 3.1 Photoactivated oxidation

**Fig. 1:**
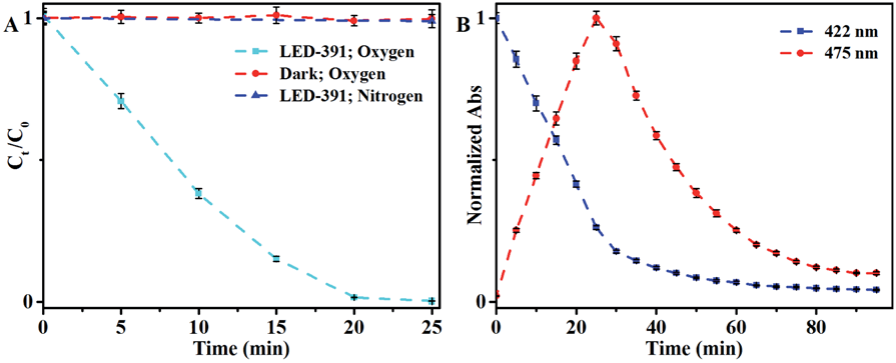
(A) Normalized concentration change of DHBP under different conditions; (B) normalized absorbance with irradiation time. Condition: LED-391, 10 °C, DHBP (1 mg mL^−1^), air. Error bar: (±) SD.

The photochromism of DHBP was first confirmed with an LED with the maximum emission at 391 nm (LED-391). The detailed procedures are given in the ESI.† The concentration of DHBP was calculated based on the calibration curve shown in Fig. S2.† The results show that DHBP is photochromic in the presence of oxygen (Fig. 1A). No change was observed without irradiation in an atmosphere with oxygen, while in an atmosphere of nitrogen, there is no change too, even with prolonged irradiation (Fig. 1A). The reaction rate is dependent on the light intensity, the temperature and the initial concentration of the reactant (Table 1 and Fig. S4†). When the LED is connected in parallel with a 10 Ω resistor, the oxidation rate decreases from 208 μg min^−1^ to 96 μg min^−1^ (Fig. S4†). After the distance from the liquid surface to the LED increased from 3.5 cm to 7 cm, the oxidation rate decreased from 208 μg min^−1^ to 74 μg min^−1^ (Fig. S4†). When the initial concentration increased from 0.5 mg mL^−1^ to 2.0 mg mL^−1^, the oxidation rate decreased from 343 μg min^−1^ to 113 μg min^−1^ (Table 1). At 10 °C, the oxidation rate of DHBP was 208 μg min^−1^ with 1 mg mL^−1^ of initial concentration.

**Table 1:** Oxidation rate of DHBP at different temperatures and concentrations^a^

	*T* (°C)				DHBP (mg mL^−1^)			
	10	20	30	40	0.5	1.0	1.5	2.0
Rate (μg min^−1^)	208	235	254	289	343	208	146	113

a Condition: LED-391, air.

The reaction process was monitored through absorbance (Fig. 1B) showed that in the first 25 min the absorbance at 422 nm decreased linearly while the absorbance at 475 nm increased correspondingly, which indicated the conversion of DHBP to BP. When all DHBP converted to BP, further irradiation caused decrease of the absorbance at 475 nm if there was enough oxygen (Fig. S5†). When the irradiation time was extended to 80 min, the absorbance at 475 nm reduced to 12% of that at 25 min. As illustrated by its mass spectrum, the product of photo-reaction of BP is a mixture (Fig. S6†).

For the synthesis purpose, when the concentration of DHBP dropped to less than 1% of the initial, the reaction was terminated by turning off the light and the product was precipitated by water. Almost all DHBP was converted to BP with high purity.

### 3.2 Autocatalysis at longer wavelengths

**Fig. 2:**
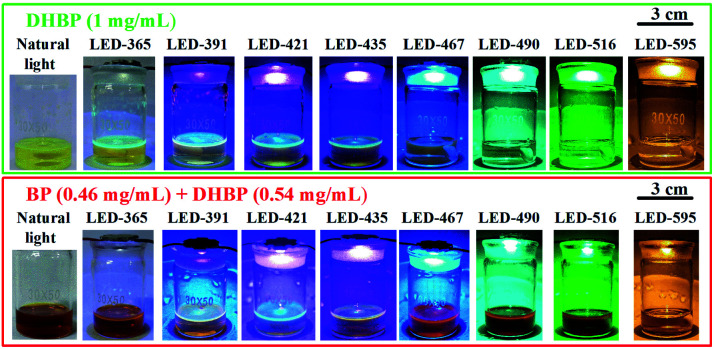
Photographs of the reaction solution under different lighting conditions.

LEDs of different color (labelled as LED-wavelength, Table S2†) were used to examine the influence of the wavelength on the photoactivated oxidation of DHBP. The photographs of the reaction solutions illuminated with different light sources were shown in Fig. 2.

**Fig. 3:**
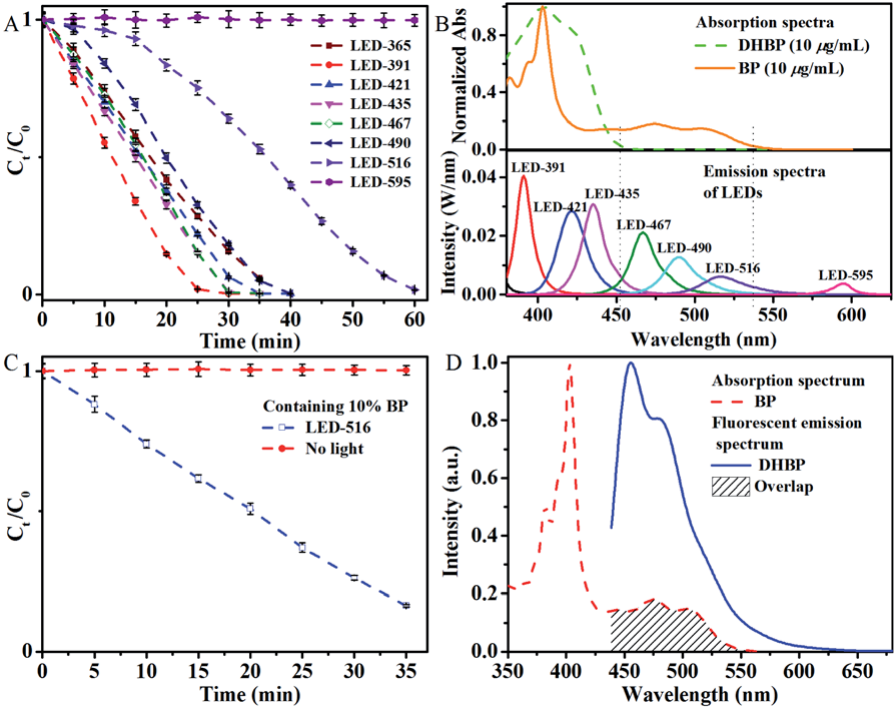
(A) Oxidation of DHBP under irradiation of LEDs with different wavelengths. Condition: temperature, 10 °C; distance, 3.5 cm; DHBP conc. 1 mg mL^−1^; atmosphere, air; (B) UV-vis absorption spectra of DHBP and BP (solvent: DMSO/ethanol = 4/1), and the emission spectra of LEDs; (C) the relative concentration of DHBP *versus* time in the presence of BP (light source LED-516); (D) normalized absorption spectrum of BP and the fluorescence emission spectrum of DHBP. Error bar: (±) SD.

The results indicate that the photoreaction is wavelength dependent (Fig. 3A). For wavelengths up to 452 nm, typical photo-chemical conversion of DHBP to BP was observed. The reaction rates are 141 μg min^−1^ (for LED-365), 208 μg min^−1^ (for LED-391), 158 μg min^−1^ (for LED-421) and 166 μg min^−1^ (for LED-425) respectively. This was the consequence of the absorption of photons by DHBP due to the overlap of these emissions with the absorption spectrum of DHBP (Fig. 3B).

However, with LEDs of 490 and 516 nm, conversion of DHBP was also observed although there is no absorption of DHBP at these wavelengths (Fig. 3B). A typical inverse sigmoidal type of the conversion curves was observed for these two wavelengths, which indicates the presence of autocatalysis. Maximum reaction rates are 172 μg min^−1^ (for LED-490) and 111 μg min^−1^ (for LED-516) respectively. As illustrated by the absorption bands of DHBP and BP (Fig. 3B), the irradiation of 516 and 490 nm are absorbed by BP but not by DHBP, which means BP can act as a kind of catalyst to convert DHBP to BP.

To verify the role of BP, 10 mol% BP was added to a DHBP solution and irradiated with LED-516, the curve became same as that irradiated with shorter wavelengths (Fig. 3C). As a contrast, the DHBP solution containing BP stayed unchanged without light irradiation. These results indicate a photo-autocatalysis of oxidation of DHBP to BP.

### 3.3 Mechanism of autocatalysis

**Fig. 4:**
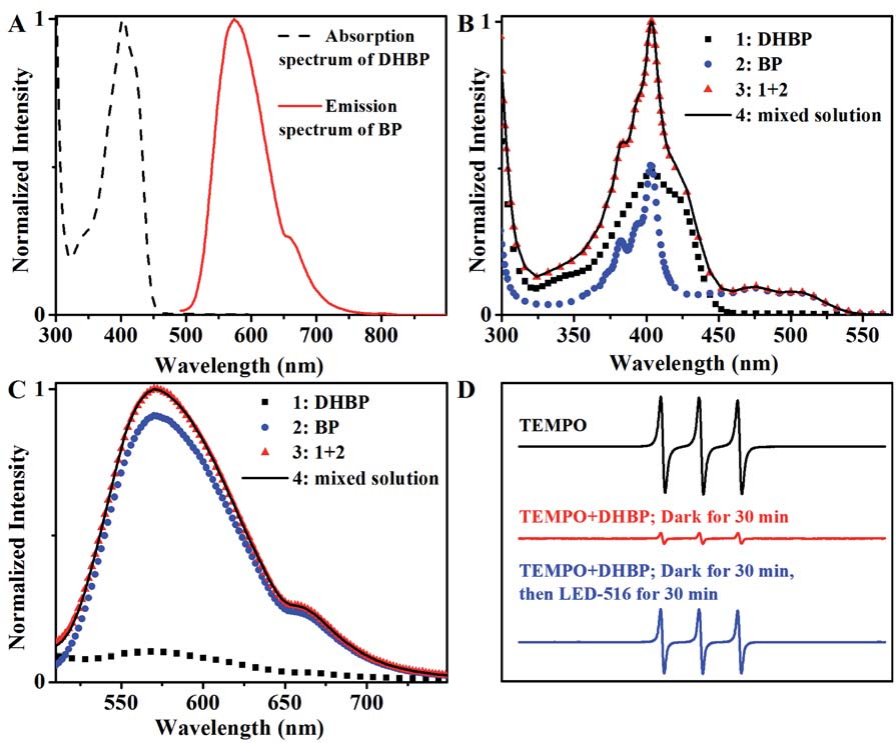
(A) Normalized spectra of DHBP (absorption) and BP (emission) at *λ*_ex_ = 402 nm; (B) absorption spectra of DHBP, BP and DHBP + BP; (C) emission spectra of DHBP, BP and DHBP + BP (*λ*_ex_ = 470 nm). Conditions: the concentration of DHBP or BP were 10 μg mL^−1^; (D) X-band EPR spectra of the TEMPO under different conditions.

There are some reports on the photoactivated autocatalysis of fluorescein derivative,^21^ α-diketone^24^ and boron-dipyrromethene (BODIPY) derivative.^25^ However, study of the effect of wavelength on the photoautocatalytic reaction is scarce. In some of the reported photoactivated autocatalysis,^22^ the autocatalytic process is derived from the Förster resonance energy transfer (FRET) between the product and the starting material, where the overlap between the fluorescence emission band of the product and the absorption band of the starting material is essential. But in this case, there was no overlap between the absorption band of DHBP and the fluorescence emission band of BP (Fig. 4A). On the contrary, the fluorescence emission band of DHBP overlaps with the absorption band of BP (Fig. 3D). The energy of BP acquired by absorption of photons of wavelengths of 490 and 516 nm cannot transfer to DHBP through a FRET process.

In addition, the spectrum of the mixture of DHBP and BP was exactly the sum of the spectra of DHBP and BP (Fig. 4B and C), which basically indicates that there is no molecular interaction between DHBP and BP either in the ground state or excited state including the charge transfer, electron transfer or the formation of exciplex.

The effect of concentration was also examined. The autocatalytic behaviour was observed at a very low concentration (10 μg mL^−1^ DHBP) (Fig. S7†), which is another evidence that no intermolecular interaction is involved.

To test if there are any oxidized products from BP act as the oxidants to convert DHBP to BP, we irradiated 5 mL of BP solution (1 mg mL^−1^) with LED-391 under ambient atmosphere until the concentration of BP dropped to 0.1 mg mL^−1^, then added 5 mg DHBP into the solution and the mixture was irradiated for another 25 min with LED-391 under a nitrogen atmosphere. The content of DHBP in the solution only decreased 1.1%, which was same as that of pure DHBP irradiated under the same condition. This result indicates that none of the photo-degraded products contributed to the conversion of DHBP to BP.

Phenazines have been successfully used as photocatalysts in atom transfer radicals polymerization^14,23^ and oxidative amidation of aromatic aldehydes,^20^ reactive oxygen species or radicals were proposed. To check if there were reactive oxygen species in this system, 2,2,6,6-tetramethylpiperidine-1-oxyl radical (TEMPO) was used as an indicator.^26–28^ As shown in Fig. 4D, the presence of DHBP largely suppressed the EPR signal of TEMPO, which was probably caused by the reduction of TEMPO radicals. However, when the mixture was radiated with LED-516 for 30 min, EPR signal resumed, indicating the formation of reactive oxygen species under the light irradiation.

### 3.4 Applications

**Fig. 5:**
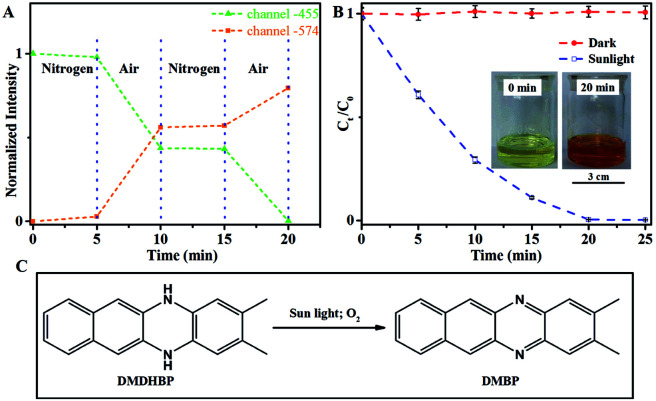
(A) Changes of fluorescent intensities of the solution in different atmosphere (LED-490); (B) the relative concentration of DHBP *versus* time. The inset photos are the reaction solutions before and after being exposed to the sunlight for 20 minutes; (C) sunlight-activated oxidation of DMDHBP to DMBP. Error bar: (±) SD.

The photoactivated autocatalytic oxidation of DHBP could be used as a sensor of the oxidizing substances (Fig. 5A). With nitrogen, only slight change of the fluorescent intensity at either 455 nm or 574 nm was observed, which indicates the slow conversion of DHBP to BP, possibly by dissolved trace amount of oxygen. When the system was filled with air, remarkable change of fluorescence in both channels was observed. When the atmosphere switched back to nitrogen, the change of fluorescence reduced to the original level again.

Sunlight can be readily used to oxidize DHBP (Fig. 5B). With sunlight, the oxidation rate was calculated to be 393 μg min^−1^ for the initial 5 min, which is 1.9 times faster than using LED-391.

The method can be extended to other molecules. 2,3-Dimethylbenzo[*b*]phenazine (DMBP) was successfully synthesized through a similar process using 5,12-dihydro-2,3-dimethylbenzo[*b*]phenazine (DMDHBP) as a raw material (Fig. 5C). A solution of DMDHBP (19.2 μmol) in the mixed solvent (5 mL) was stirred in air under sun light. The reaction was monitored by recording the fluorescent intensity of the diluted solution. When the fluorescence intensity at 455 nm fell to 1% of the initial value, an excess amount of water was added to the reaction solution to obtain precipitation. The resulting precipitate was separated, washed with water and dried. DMDHBP was almost completely converted into DMBP.

## 4. Conclusions

Photochemical oxidation of DHBP was systemically evaluated with LEDs of the central emission ranging from 365–595 nm as the light sources. The photoactivated autocatalytic oxidation of DHBP with radiation of cyan to green light was confirmed. Typical sigmoidal curves of the conversion were observed by irradiation of reaction solution with LEDs with central emissions at 490 nm and 516 nm. Because DHBP has virtually no absorption in this range of wavelength, BP is therefore responsible for the catalysis. It illustrates that photochemical reaction may occur under the light irradiation that the reactants do not absorb. A reaction rate of 208 μg min^−1^ was achieved with the inexpensive LED and it facilitates the reaction. Sunlight can be also used to achieve green synthesis of BP, and the oxidation rate of 1 mg mL^−1^ DHBP was 393 μg min^−1^ in a 30 mm diameter glass vessel. With this photochemical process, no metal ion containing oxidant or catalyst is needed, which is advantageous for metal-free compounds used for organic semiconductor devices. The reaction process can be easily monitored with the fluorescence, so nearly quantitative conversion of H_2_-NHAs to NHAs can be achieved. In addition, because of its oxygen activation capability, relatively inert chemical property and fluorescence emission of BP, this photoactivated autocatalysis process may find its usage in residual oxygen detection and phototherapy with cyan to green light.

## Conflicts of interest

There are no conflicts to declare.

## Supplementary Material

RA-010-D0RA01495H-s001
