# Supplementary material for: Wavelength selective photoactivated autocatalytic oxidation of 5,12-dihydrobenzo[b]phenazine and its application in metal-free synthesis
Source: RSC Adv. 2020 Mar 9;10(17):9949–54. doi: 10.1039/d0ra01495h (PMC9050338; doi:10.1039/d0ra01495h)
Supplement: RA-010-D0RA01495H-s001 [file RA-010-D0RA01495H-s001.pdf]

Electronic Supplementary Material (ESI) for RSC Advances.
This journal is © The Royal Society of Chemistry 2020

Electronic Supplementary Information

Wavelength selective photoactivated autocatalytic oxidation of 5, 12-dihydrobenzo[b]phenazine and its application in metal-free synthesis

Lei Bian,[a] Jie Ma,[a,b] Xiaotong Feng,[a] Yuanhang Wang,[a] Lizhi Zhao,[a] Lei Zhao,[a] Xiayan Wang,[c] Guangsheng Guo[c] and Qiaosheng Pu*,[a]

[a]State Key Laboratory of Applied Organic Chemistry, Key Laboratory of Nonferrous Metals Chemistry and Resources Utilization of Gansu Province, College of Chemistry and Chemical Engineering, Lanzhou University, Lanzhou, 730000, China.

[b]College of Chemical Engineering and Technology, Tianshui Normal University, Tianshui, 741001, China.

[c]Department of Chemistry and Chemical Engineering, Beijing University of Technology, Beijing, 100124, China.

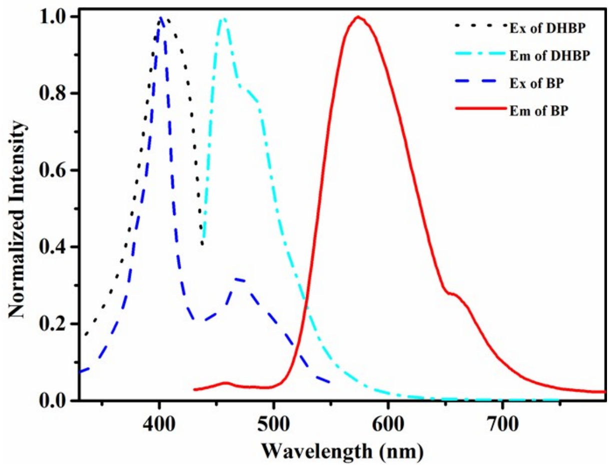

Figure (S1) Normalized emission spectrum and excitation spectrum of DHBP (1 μg/mL) and BP (1 μg/mL). Solvent: A.

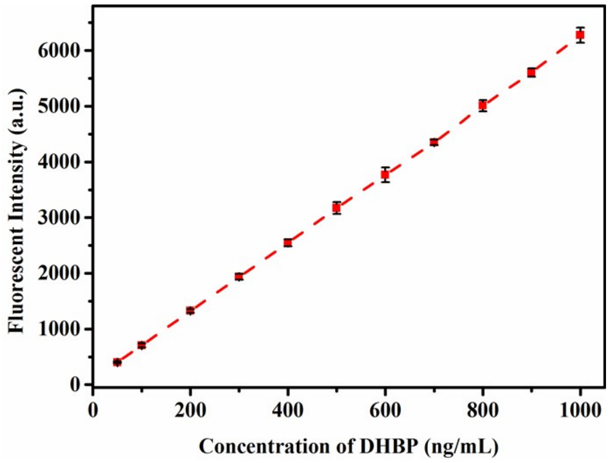

Figure (S2) Plots of fluorescence intensity (λ_{ex} = 408 nm / λ_{em} = 455 nm) versus the concentration of DHBP. Error bar: (±) SD.

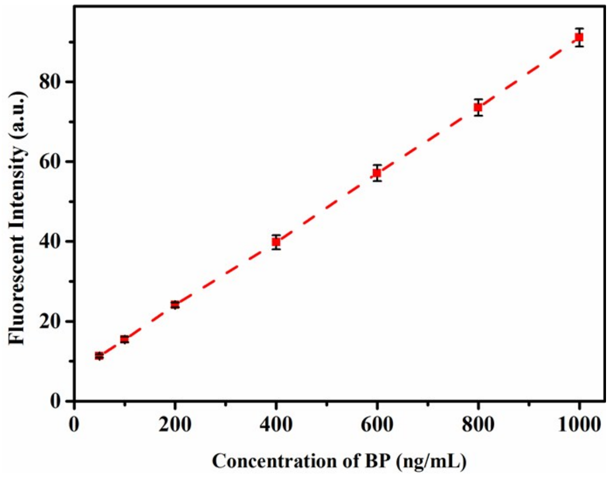

Figure (S3) Plots of fluorescence intensity (λ_{ex} = 470 nm / λ_{em} = 576 nm) versus the concentration of BP. Error bar: (±) SD.

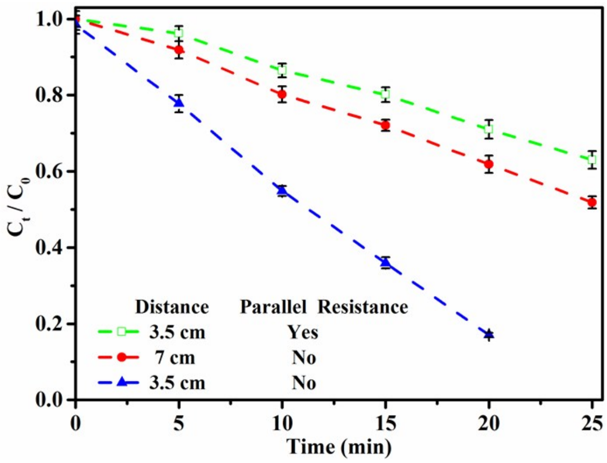

Figure (S4) Plots of C_t/C_0 versus time illuminated by LED-391 under different light intensities. Error bar: (±) SD. Experimental condition: LED-391, 10 °C, DHBP (1 mg/mL; 5mL), air, the parallel resistor: 10 Ω.

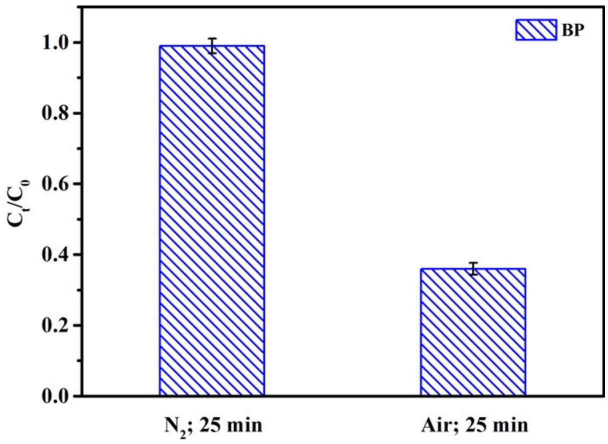

Figure (S5) BP's relative concentrations at different atmospheres after being illuminated by LED-516 for 25 minutes. Error bar: (±) SD.

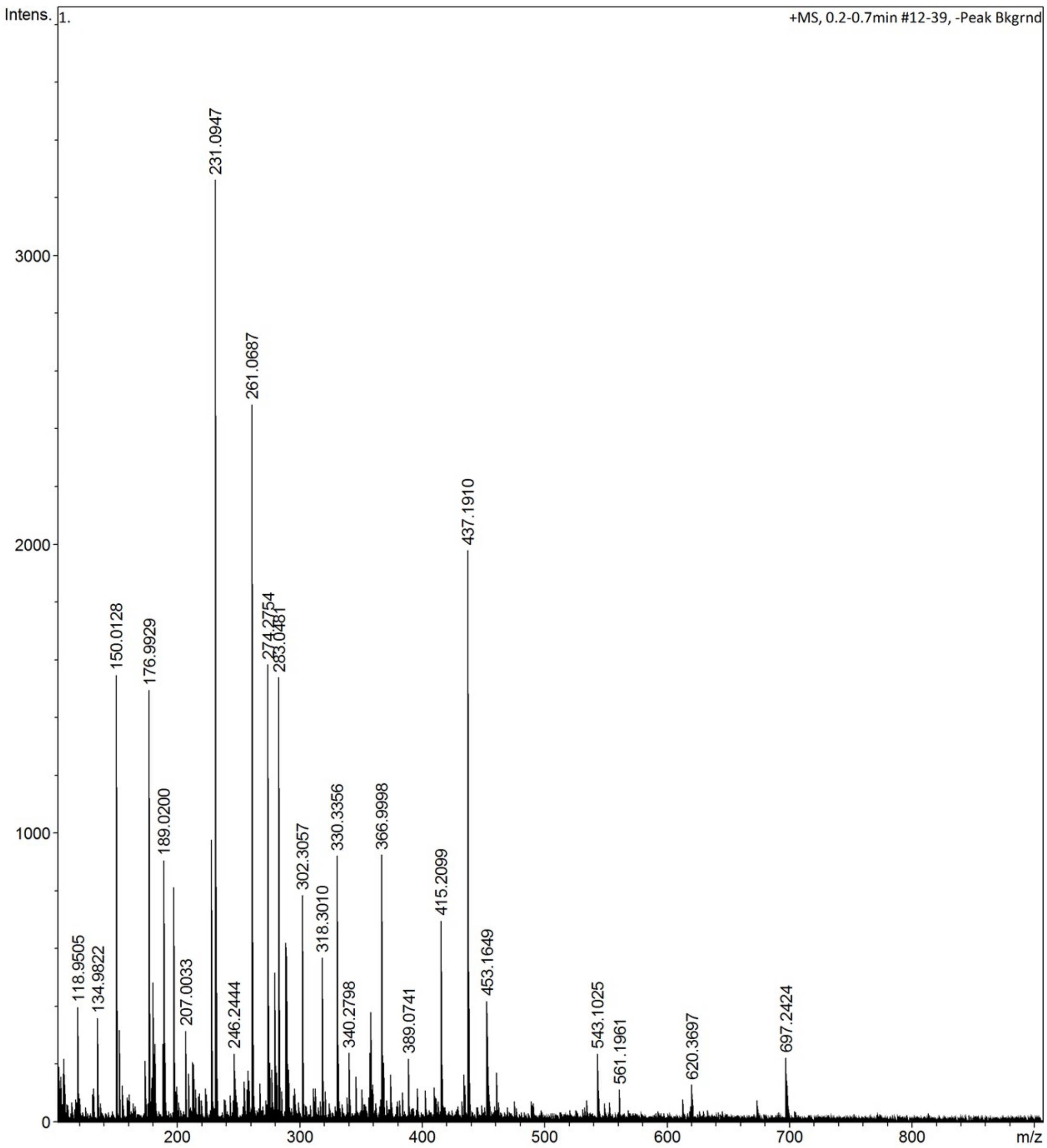

Figure (S6) Mass spectrometry of BP solution after being illuminated by LED-391 for 65 minutes. Experimental condition: LED-391, 10 °C, BP (the initial concentration was 1 mg/mL; 5mL), air.

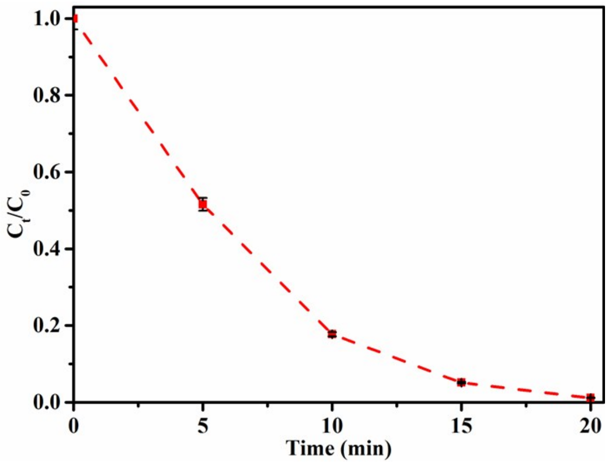

Figure (S7) Oxidation of DHBP in the mixed solution of DHBP and BP. Experimental conditions: the solvent was a mixture of DMSO and ethanol with volume ratio of 4:1, the concentration of DHBP or BP were 10 μg/mL and LED-516 was used as light source. Error bar: (±) SD.

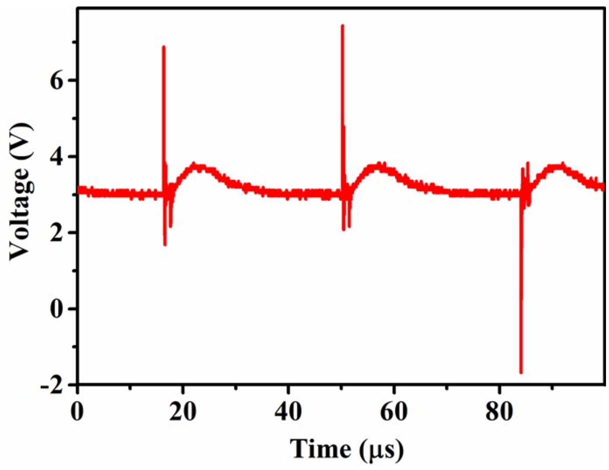

Figure (S8) Plots of voltage versus time when the LED-391 is working.

Table S1. Working voltages of LEDs in photochemical oxidation.

LED	365	391	421	435	467	490	516	595
Voltage (V)	3.17	3.19	3.16	3.11	2.87	2.85	2.93	2.16

Table S2. Operational condition and characteristic of the light sources.

LED	365	391	421	435	467	490	516	595
Voltage (V)	3.33	3.20	3.21	3.15	2.93	3.01	3.12	2.25
Current (A)	0.321	0.321	0.321	0.321	0.321	0.321	0.321	0.321
Radiation power (W)	--[a]	0.617	0.685	0.649	0.504	0.372	0.228	0.067
Radiation photon number (μmol/s)	--[a]	2.03	2.43	2.38	1.98	1.54	0.99	0.33

[a] The emission wavelength of LED-365 is beyond the measurement range of the instrument.

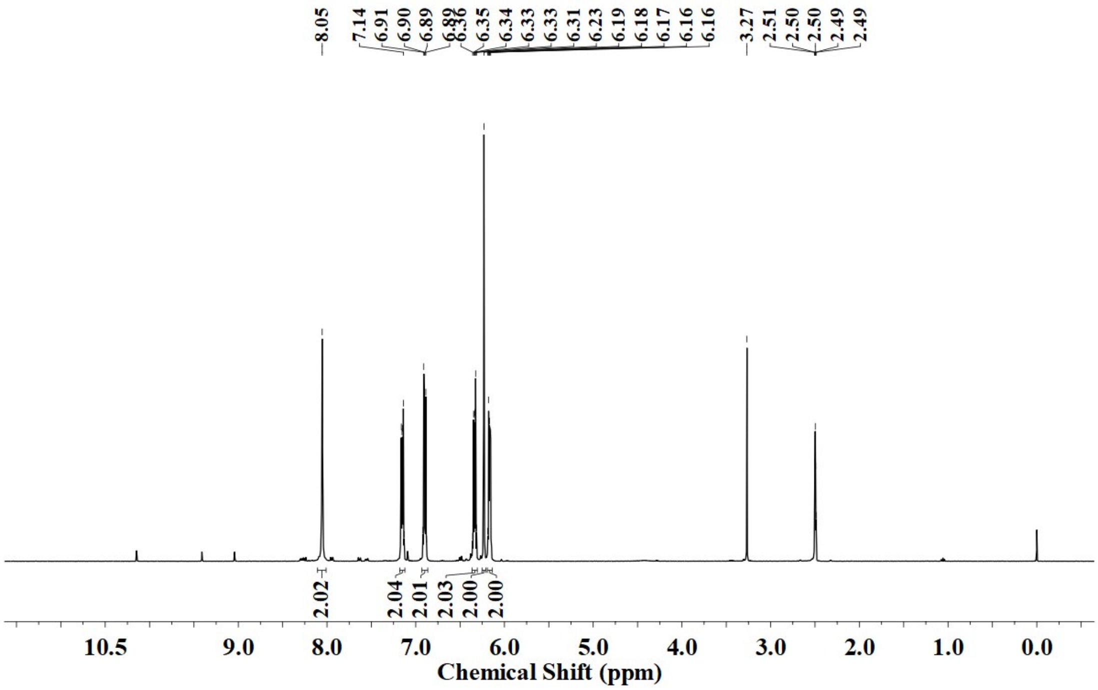

Figure (S9) ^{1}H NMR of DHBP in DMSO-d6.

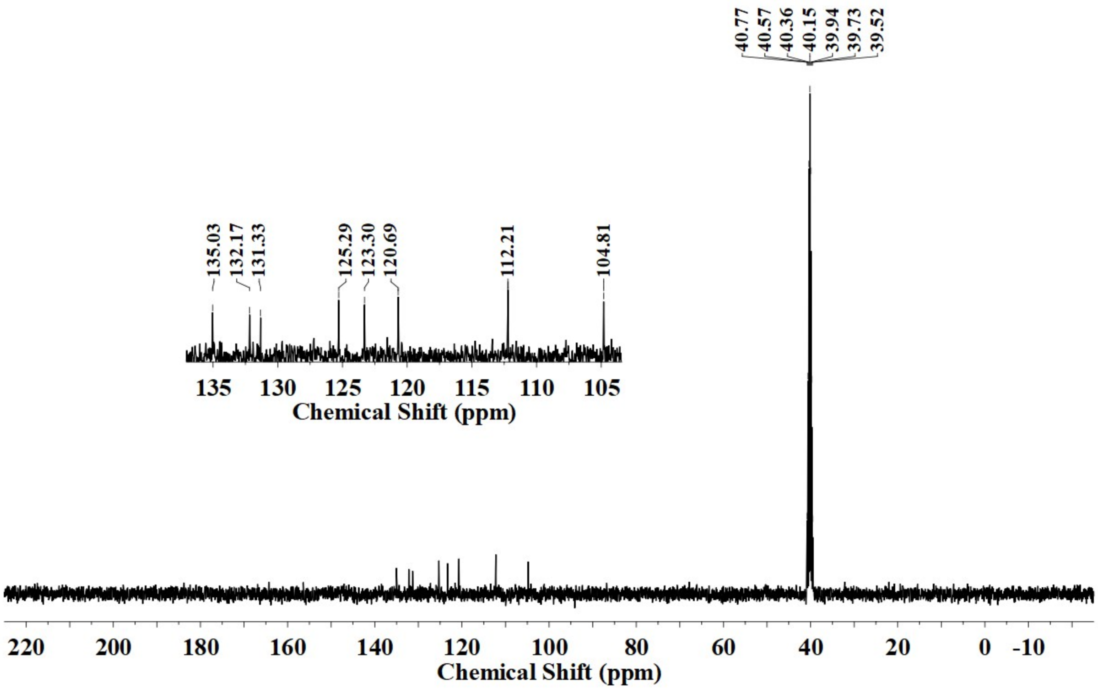

Figure (S10) ^{13}C NMR of DHBP in DMSO-d6.

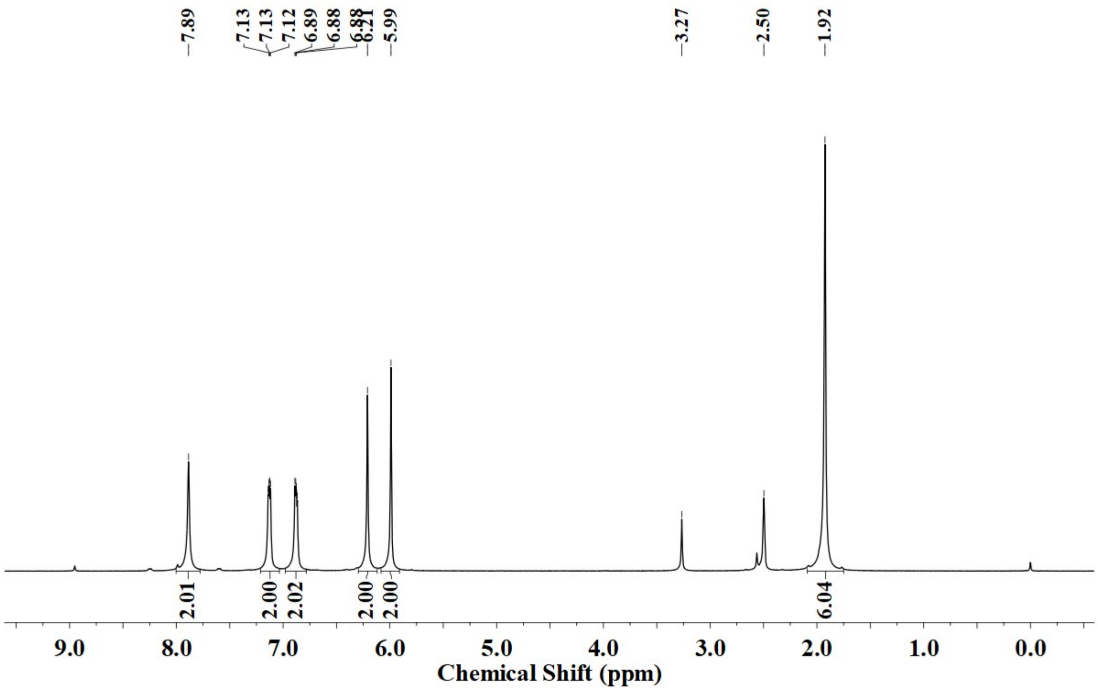

Figure (S11) ^{1}H NMR of DMDHBP in DMSO-d6.

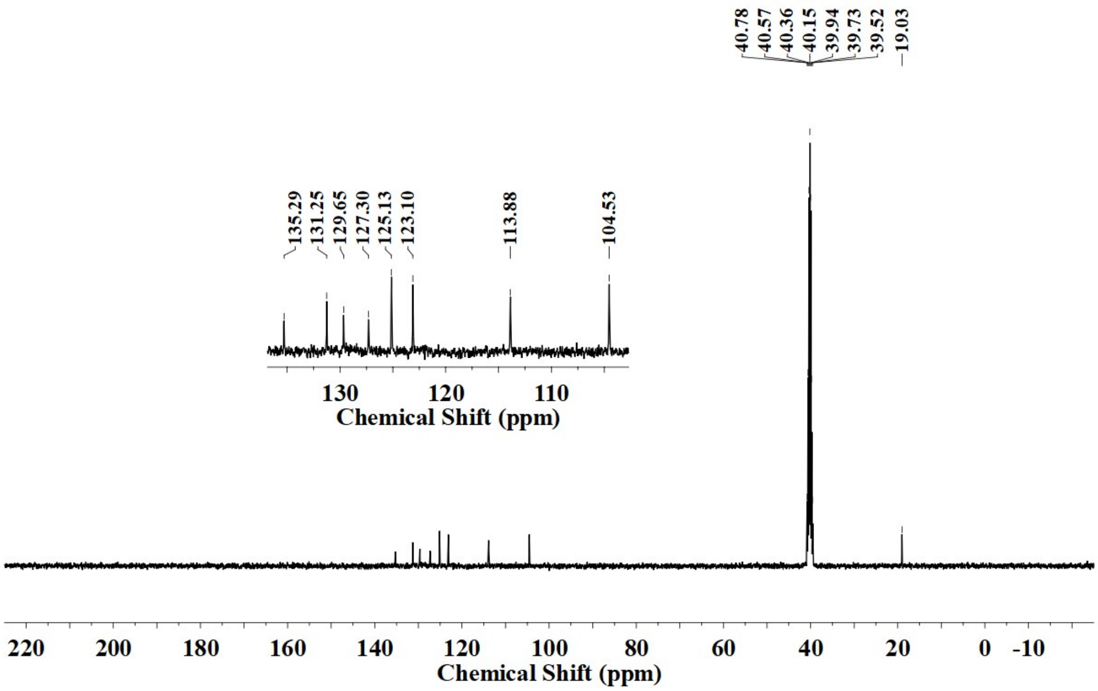

Figure (S12) ^{13}C NMR of DMDHBP in DMSO-d6.

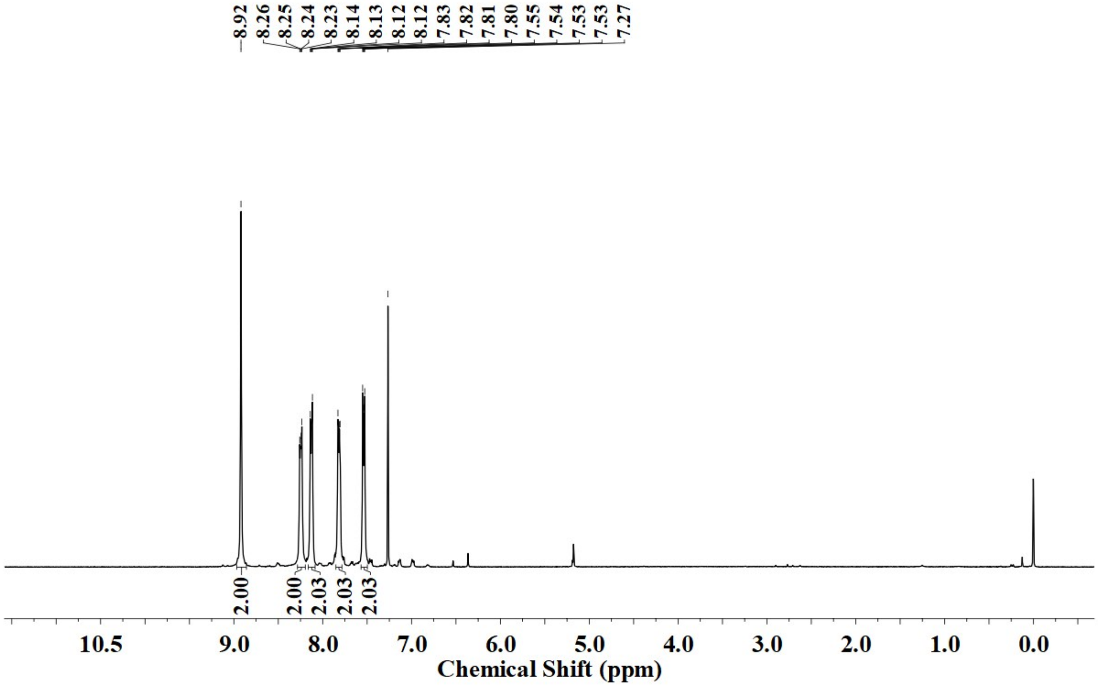

Figure (S13) ^{1}H NMR of BP in $CDCl_3$.

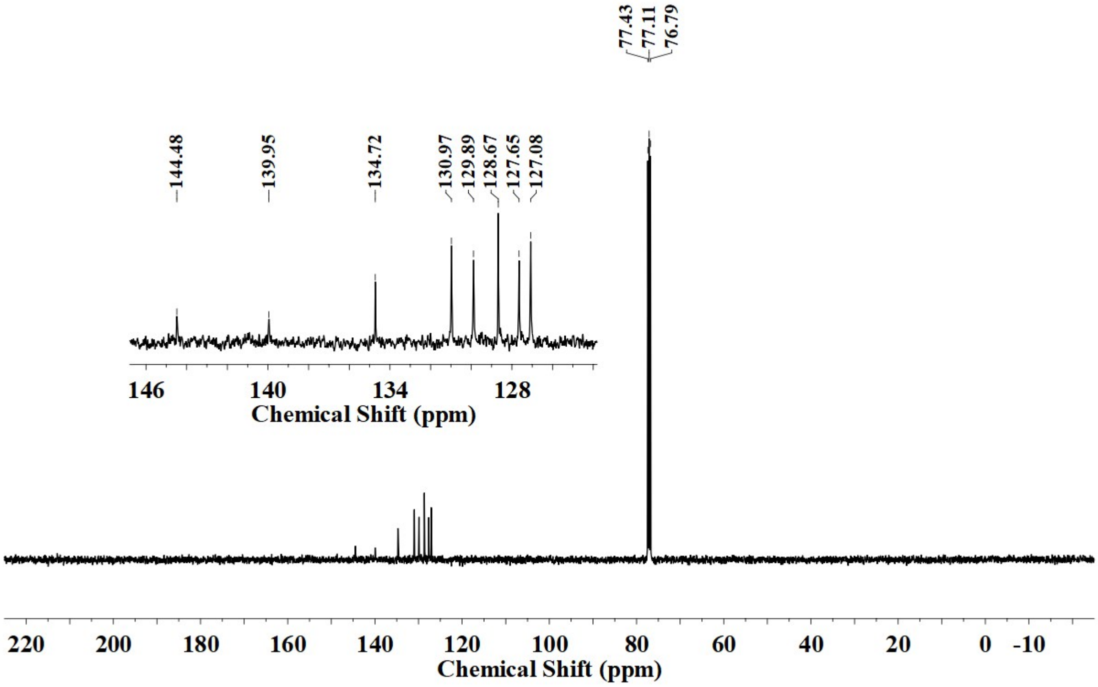

Figure (S14) ^{13}C NMR of BP in $CDCl_3$.

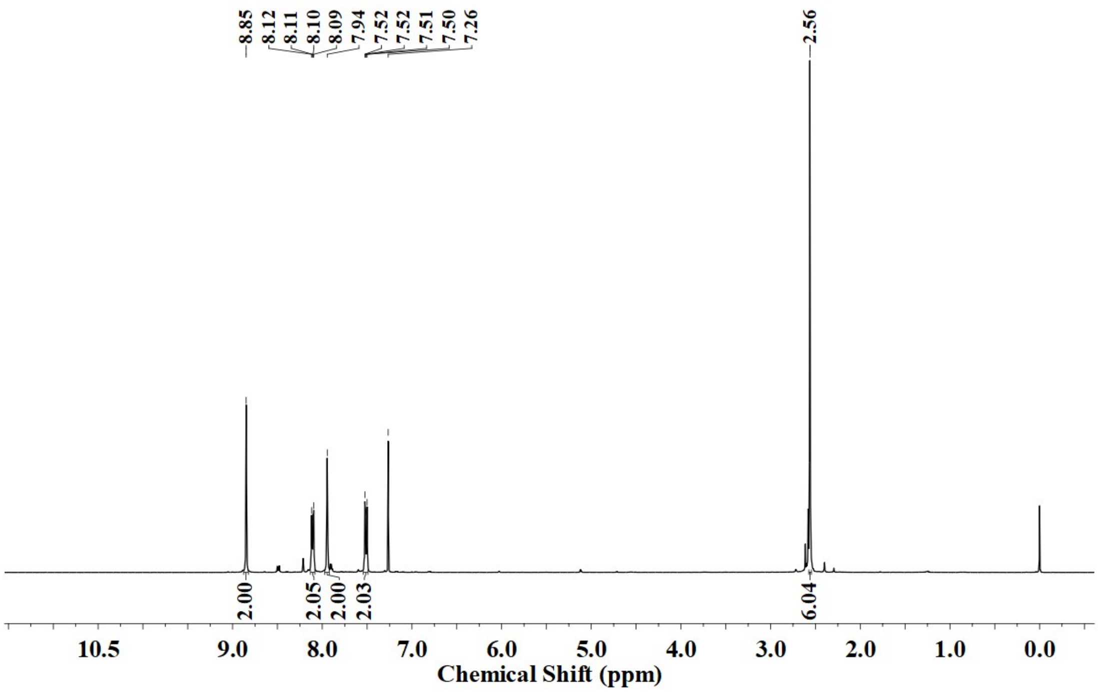

Figure (S15) ^{1}H NMR of DMBP in $CDCl_3$.

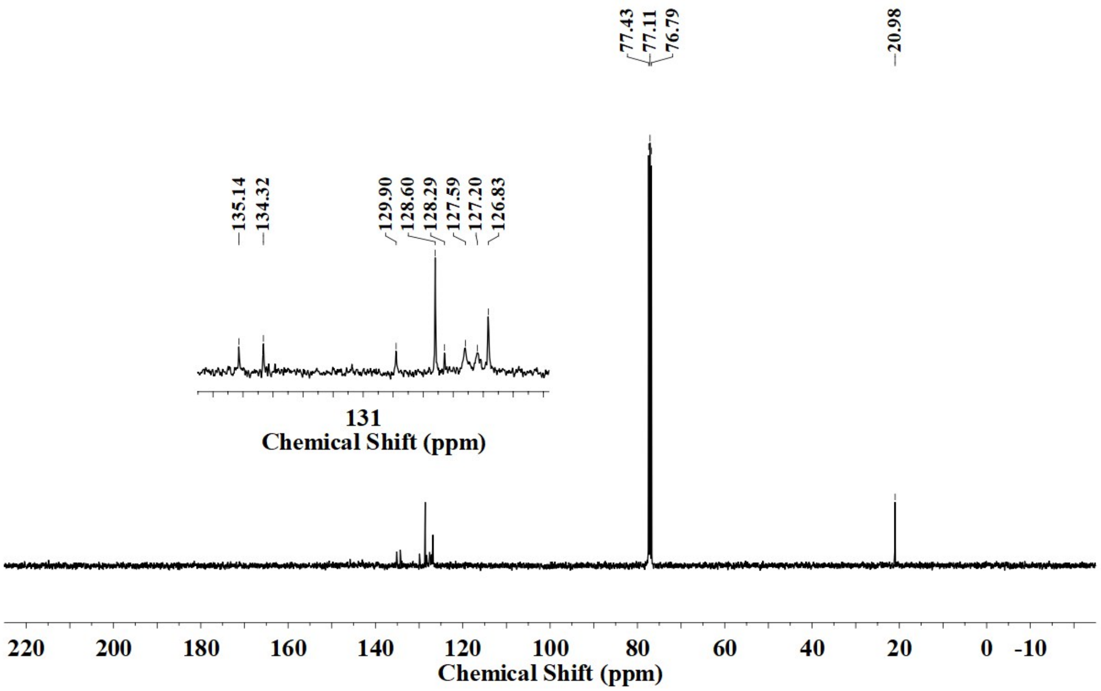

Figure (S16) ^{13}C NMR of DMBP in $CDCl_3$.
